# The MARECA (national study of management of breast cancer locoregional recurrence and oncological outcomes) study: protocol for a prospective, multicentre cohort study

**DOI:** 10.1097/SP9.0000000000000018

**Published:** 2024-01-26

**Authors:** Sue M. Hartup, Jenna L. Morgan, Vinton WT Cheng, Peter A. Barry, Ellen Copson, Ramsey I. Cutress, Rajiv Dave, Beatrix Elsberger, Patricia Fairbrother, Brian Hogan, Kieran Horgan, Cliona C. Kirwan, Stuart A. McIntosh, Rachel L. O’Connell, Neill Patani, Shelley Potter, Tim Rattay, Lisa Sheehan, Lynda Wyld, Baek Kim

**Affiliations:** a The Breast Unit at the Leeds Cancer Centre, St James’s University Hospital, Leeds Teaching Hospitals NHS Trust, Leeds; b Division of Clinical Medicine, University of Sheffield School of Medicine and Population Health, Beech Hill Road, Sheffield; c Department of Breast Surgery, The Royal Marsden NHS Foundation Trust, Sutton, Surrey; d The Institute of Cancer Research; e Department of Breast Surgery, University College London Hospitals NHS Foundation Trust; f Trustee, Independent Cancer Patients Voice, London, UK; g Aberdeen Breast Unit, Aberdeen Royal Infirmary, Foresterhill, Aberdeen; h The Nightingale Breast Cancer Centre, Wythenshawe Hospital; i Division of Cancer Sciences, School of Medical Sciences, Faculty of Biology, Medicine and Health, University of Manchester, Oglesby Cancer Research Building, Manchester Cancer Research Centre, Manchester; j Patrick G Johnston Centre for Cancer Research, Queen’s University Belfast, Belfast; k Somers Cancer Research Building, University of Southampton and University Hospital Southampton, Southampton; l Translational Health Sciences, Bristol Medical School, Learning and Research Building, Southmead Hospital; m Bristol Breast Care Centre, North Bristol NHS Trust, Bristol; n Leicester Cancer Research Centre, Clinical Sciences Building, University of Leicester, Leicester Royal Infirmary; o Department of Breast Surgery, University Hospitals of Leicester NHS Trust, Leicester; p Wessex Deanery, Southern House, Otterbourne, Winchester, UK

**Keywords:** breast cancer, locoregional recurrence

## Abstract

**Background:**

Despite a UK 5-year breast cancer survival rate of 86.6%, patients may develop breast cancer recurrence within the same breast after breast conserving surgery, as well as in the remaining skin or chest wall after mastectomy or in the ipsilateral lymph glands. These recurrences, collectively termed locoregional recurrence (LRR), occur in around 8% of patients within 10 years of their original diagnosis. Currently, there is a lack of robust information on the presentation and prevalence of LRR with no UK-specific clinical guidelines available for the optimal management of this patient group. Additionally, there is a need to identify patterns of LRR presentation and their progression, which will enable prognostic factors to be determined. This will subsequently enable the tailoring of treatment and improve patient outcome.

**Methods:**

The MARECA study is a prospective, multicentre cohort study recruiting patients diagnosed with breast cancer LRR +/- associated distant metastases. Over 50 UK breast units are participating in the study with the aim of recruiting at least 500 patients over a recruitment period of 24 months. The data collected will detail the tumour pathology, imaging results, surgical treatment, radiotherapy and systemic therapy of the primary and recurrent breast cancer. Study follow-up will be for up to 5 years following LRR diagnosis to determine subsequent oncological outcomes and evaluate potential prognostic factors.

**Discussion:**

This study will address the current knowledge gap and identify subgroups of patients who have less successful treatment outcomes. The results will determine the current management of LRR and the prognosis of patients diagnosed with breast cancer LRR +/- distant metastases in the UK, with the aim of establishing best practice and informing future national guidelines. The results will direct future research and inform the design of additional interventional trials and translational studies.

Highlights

- Breast cancer locoregional recurrence (LRR), occurs in around 8% of patients within 10 years of their original diagnosis.
- There is a lack of robust information on the presentation and prevalence of locoregional recurrence with no UK-specific clinical guidelines available for the optimal management of this patient group.
- The MARECA study will help to determine oncological outcomes and evaluate potential prognostic factors, subsequently enabling the tailoring of treatment, improved patient outcomes and aid the production of national guidelines.

## Background

Breast cancer is diagnosed in ~55 000 women per year in the UK^1^ and 5-year survival for all breast cancer patients is favourable at 86.6% due to advances in systemic treatment and implementation of the NHS breast screening programme^2^. Despite this high survival rate, a proportion of patients are diagnosed with a breast cancer recurrence within the conserved breast, ipsilateral skin or chest wall following mastectomy, or in the ipsilateral axillary, supraclavicular, infraclavicular, or internal mammary lymph nodes. These cancer recurrences are collectively termed locoregional recurrence (LRR).

In-breast local recurrence after breast conserving surgery (BCS) is thought to be due to the growth of previously undetected microscopic tumour foci, which may present when the patient presents with new symptoms or more commonly discovered on follow-up (surveillance) mammography. Local recurrence after mastectomy may present as subcutaneous nodules on the chest wall with dermal lymphatic involvement, which may be associated with worse prognosis^3^. The Early Breast Cancer Trialists’ Collaborative Group (EBCTCG) meta-analyses^4,5^ suggest that patients diagnosed with LRR after BCS and radiotherapy may have a better subsequent prognosis than those patients who initially received mastectomy. However, an adequately powered study is required to examine the prognostic effect of initial breast cancer surgery (BCS and radiotherapy vs. mastectomy) on patients diagnosed with LRR without distant metastasis (DM).

Furthermore, when patients are diagnosed with a LRR, there can be uncertainty as to whether this is a true recurrence (TR) or a new primary (NP) breast cancer. Currently, there is no standardised classification system and therefore, various definitions have been reported in the literature^6^. TR may be defined as cancers with a similar molecular receptor profile (ER; oestrogen receptor / PR; progesterone receptor / HER2) and histological subtype (Ductal/Lobular/ductal carcinoma in situ) when compared to the originally treated cancer. For patients who have had previous BCS, the tumour may be classified as TR if the recurrence location is in the same quadrant of the breast as the original tumour^7^. Patients diagnosed with NP are thought to have a better prognosis when compared to patients diagnosed with a TR^8,9^. However, comparison of tumour morphology, histological subtype, or anatomical location does not enable accurate differentiation between a TR and a NP. Genomic analysis enables a more precise distinction between a TR and a NP^10^. Pan-genomic analysis of paired samples of primary tumour with LRR has been studied in 22 patients with results suggesting its potential clinical utility^11^. However, further studies with larger sample sizes are required.

LRR rates are thought to be declining. A population-based study in the Netherlands^12^ demonstrated that the 5-year local recurrence rate after BCS had fallen from 9.8 to 3.3% (comparison of patients diagnosed between 1988 and 1998 versus 2006–2010). Low 5-year local recurrence rates (<3%) after BCS have also been reported in contemporary trials such as IMPORT-LOW^12^ and FAST-Forward^13^. However, LRR can present in numerous anatomical locations and may occur at any time after the original cancer treatment. A German registry-based study^12^ followed up patients diagnosed with breast cancer between 1999 and 2009 and reported that 10-year cumulative LRR incidence was as high as 8%, however.

In 2012, the UK National Cancer Registration and Analysis Service (NCRAS) and the National Cancer Intelligence Network (NCIN) reported a pilot audit project http://www.ncin.org.uk/view?rid=1043 for patients diagnosed with recurrent and metastatic breast cancer. The pilot involved 15 UK breast units (out of 144) who participated for 6 months in 2011. They identified 137 patients with LRR only and 114 patients with both locoregional and distant recurrences. However, this project did not investigate patient and tumour characteristics or evaluate the patient’s initial cancer treatment. Furthermore, there was a lack of detailed analysis of treatments received by the patients diagnosed with LRR or evaluation of any variation in patient management between participating units. Most importantly, these patients were not followed up to determine survival outcomes.

Currently, there are no UK-specific guidelines for the management of breast cancer LRR. The American National Comprehensive Cancer Network (NCCN) clinical practice guidelines in oncology https://www2.tri-kobe.org/nccn/guideline/breast/english/breast.pdf (BINV-19) provides recommendation as to how patients with LRR should be managed in terms of staging investigations to detect for the presence of DM, type of LRR resection surgery and types of adjuvant treatments to be offered. However, due to the relative lack of high-quality evidence, the guideline acknowledges uncertainties including optimal axillary management, the role of LRR resection in the presence of DM, and which staging investigations should be utilised. A multidisciplinary approach is advocated to optimise patient outcomes. However, to enable tailored treatment recommendations for individual patients and achieve optimal outcome, further research is needed to gather data on tumour characteristics and treatment details of the initial and recurrent cancer.

The number of patients diagnosed with LRR in each UK breast unit per year is relatively small^14^. Therefore, a national collaborative multicentre study is required. Given the concerns about under-reporting of breast cancer LRR to the UK national cancer registries https://www.nabcop.org.uk/content/uploads/2020/07/NABCOP-2020-Annual-Report-V1_high-res.pdf, retrospective analysis using existing routine data source (e.g. Hospital Episode Statistics) is unlikely to provide data on patient management that is reflective of current national practice. Well-established national breast surgery research collaboratives^15–17^ in the UK now have the capacity to generate meaningful prospective datasets that describe current national practice. Therefore, such a national collaborative can be utilised to gather a large prospective cohort of patients diagnosed with LRR with the aim of identifying risk factors for breast cancer LRR, and describe current treatments pathways and patient outcomes.

## Methods and analysis

The aim of the MARECA study is to determine the current management and prognosis of patients diagnosed with breast cancer LRR in the participating UK breast units with the aim of establishing best practice and informing future national guidelines.

The objectives are as follows:

1. Undertake a national survey^14^ of UK breast units in order to establish the current stated practice of breast MDTs regarding the management of patients with breast cancer LRR and identify any potential variation in practice.
2. Establish a prospective cohort of breast cancer patients diagnosed with LRR +/- DM in order to:

- Describe current management including:

- Use of radiological staging investigations and the proportion of patients found to have DM at presentation.
- Surgical management including the use of a repeat BCS and sentinel lymph node biopsy.
- Use of systemic therapies and radiotherapy used to treat LRR.

- Investigate risk factors associated with LRR development including those related to the primary breast cancer and its management.

- Determine the following oncological outcomes at 3 and 5 years, and evaluate prognostic factors;

- Disease free survival (DFS) for patients diagnosed with LRR without associated DM at presentation.
- Progression free survival (PFS) for patients diagnosed with LRR with associated DM at presentation.
- Overall survival (OS) for patients diagnosed with LRR with or without associated DM at presentation.

- Stratify LRR as a TR or a NP breast cancer using existing clinical classification systems^7–9^ and explore if this distinction affects oncological outcomes.

To determine the feasibility of designing a future translational study. Patients enroled in the study will be invited to provide optional consent for permission to access archival tissue samples of both the primary cancer and the LRR. This would permit comparison of clinical and genomic classification of LRR (TR or NP) against patient survival outcome.

### Study endpoints

Primary endpoint:

DFS following LRR resection in patients who present without DM, stratified according to the type of surgery performed for the primary breast cancer (BCS and radiotherapy vs. mastectomy).

Secondary endpoints:

- PFS in patients who present with LRR with DM.
- OS in patients with LRR +/- DM at presentation.

### Study design

This is a prospective, multicentre cohort study of patients diagnosed with LRR +/- DM with an evaluation of the oncological outcomes of the study cohort at 3 and 5 years. This will be a national research study in collaboration with the Association of Breast Surgery (ABS) Academic and Research committee, the Mammary Fold Academic and Research Collaborative (MFAC) committee and the UK Breast Cancer Trainees Research Collaborative Group (BCTRCG). The study has been adopted on to the National Institute for Health and Care Research (NIHR) clinical research network portfolio enabling research nurses at the participating sites to recruit study participants. The study has also been registered on the NIHR Associate Principal Investigator scheme.

The first phase of the study, the MARECA study national practice questionnaire (NPQ), consisted of scenario-based questions for the breast cancer multidisciplinary teams (MDTs) at the participating centres. The published results^14^ of the NPQ documented the current stated practice of breast cancer LRR management by MDTs, and captured data on practice variations and areas of uncertainty in patient management.

The second phase of the study, the national prospective cohort study, aims to recruit at least 500 women and men newly diagnosed with breast cancer LRR across 50 UK breast units. Recruitment is over a 24 months period. The study has been approved by the London - Brighton & Sussex Research Ethics Committee (21/PR/1128), with the first MREC approval date 07/09/2021 and is a registered clinical trial (ISRCTN11908093). The study recruited its first study participant in January 2022.

### Study setting

We will identify and recruit study participants from breast services at NHS hospitals in all four nations of the UK.

### Study participants

Female or male patients who were previously treated for breast cancer with curative intent and who have recently (within 6 months) been diagnosed with a breast cancer LRR +/- DM. Full inclusion and exclusion criteria are shown in Tables 1 and 2. For the purpose of this study, LRR is defined as a histological diagnosis of invasive breast cancer or ductal carcinoma in situ in the ipsilateral breast (if applicable)/skin/chest wall/regional nodes (axilla/internal mammary/supraclavicular/infraclavicular) following previous breast cancer treatment with curative intent.

**Table 1:** Inclusion criteria.

Inclusion criteria
• Female or male patients more than 18 years old
• Treated for previous unilateral or bilateral breast cancer (invasive cancer including all histological subtypes as well as ductal carcinoma in situ) with curative intent
• No previous evidence of distant metastatic disease
• Recently (within the last 6 months) diagnosed with new ipsilateral breast cancer LRR (biopsy proven invasive cancer including all histological subtypes or ductal carcinoma in situ) +/- distant metastasis
• Able to provide written informed consent
• A minimum of 3 months interval between the resection surgery for the original cancer and the diagnosis of LRR. There will be no maximum interval time period

**Table 2:** Exclusion criteria.

Exclusion criteria
• Patients where the new breast cancer diagnosis is in the contralateral breast
• For patients who present with new bilateral breast cancer, the side with no previous cancer will be excluded
• Patients diagnosed with distant metastatic disease with no evidence of LRR
• Patients diagnosed with angiosarcoma
• Patients with previous history of non-breast cancer treatment that was noncurative in intent
• Patients who have had previous ipsilateral surgery for atypia, benign conditions, or phyllodes tumour and other breast sarcomas AND no previous ipsilateral primary breast cancer resection
• Patients under 18 years old
• Patients lacking capacity to provide written informed consent

### Study processes

Eligible patients recently (within the last 6 months) diagnosed with LRR will be identified by healthcare professionals directly involved in the patient’s care:

1. at breast MDT meetings;
2. in breast surgery and oncology clinics;
3. by screening patent records.

The approach will be undertaken via two routes:

1. An appropriately trained member of the team (as per the delegation log), at each unit will approach eligible patients and discuss the option of study participation and provide a study patient information sheet (PIS).
2. Identified patients may also be approached using the approved invite letter, which is posted to patients along with the PIS.

The completion of the consent form may occur during the patient’s scheduled hospital visits. In addition, patients may also consent by post following invite in person or having been approached by the approved invite letter. All study participants are invited to consider consenting to an optional tissue sub-study. This does not involve the collection of any new tissue, but requests participants’ permission to collect pre-existing samples from their original cancer and recurrence. A translational tissue sub-study is planned and will be based on the uptake rate of this optional consent.

### Study data collection

Data regarding tumour characteristics, investigations performed and treatment details for the patient’s original and recurrent cancer will be collected. Data collection will involve recording patient data that are routinely collected as part of standard clinical practice. Study data will be collected and managed using REDCap^18,19^ (Research Electronic Data Capture) electronic data capture tools The required data fields for the MARECA study prospective cohort study are summarised in the protocol appendices (Supplementary Data File, Supplemental Digital Content 1, http://links.lww.com/JS9/B670).

**Figure 1:**
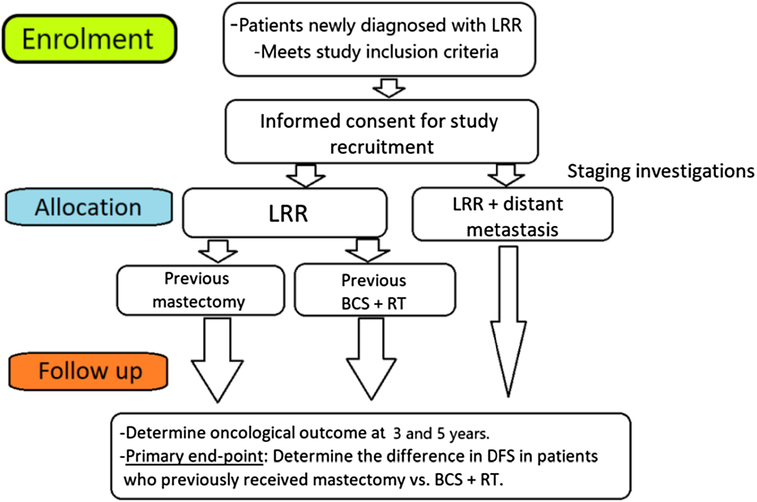
MARECA study flowchart.

The collection of oncological outcome data at 3 and 5 years (Fig. 1) post diagnosis will enable the determination of the DFS, PFS and OS of the study cohort. Data collection will include the patient’s survival status, and whether death was related to breast cancer or not, and the date of death. Data on the occurrence of any further LRR or DM, and respective dates will also be collected. To gather these data, the participating units will access their hospital electronic database and hence no direct patient contact is anticipated as part of the study procedure.

### Anticipated recruitment and sample size

Out of 144 breast units in the UK, 50 units have agreed to participate in the MARECA study. Based on the MARECA study NPQ results^14^, we would anticipate each unit to recruit 5 to 10 patients over 12 months. Therefore, the study aims to recruit at least 500 patients diagnosed with LRR +/- DM over 24 months. The study’s primary endpoint is to determine whether patient prognosis after their LRR diagnosis (without DM) differs according to the type of breast cancer surgery (mastectomy versus BCS and radiotherapy) for the original cancer. To determine the primary endpoint, logistic regression analysis was used to determine the effect of treatment type (mastectomy versus BCS and radiotherapy for the original cancer) on DFS by adjusting for other covariates (including tumour stage, node stage, tumour receptor status and tumour grade). This analysis determined that the sample size for each group would be 165 patients. Therefore, a subset of 330 patients diagnosed with LRR (without DM) will require recruiting to the study to determine the study’s primary endpoint. This falls within the minimum patient recruitment target of 500 patients.

As of 11/12/23, the study has recruited 684 patients and hence exceeded the minimum recruitment target of 500 patients. During the recruitment process, we identified that the number of patients treated with BCS for the original cancer was higher than those treated with mastectomy for the original cancer. Therefore, once we reached the overall recruitment target of 500 patients earlier in 2023, the decision was made to continue recruitment and thereby enable the minimum number of patients in each treatment arm to be reached to determine the study’s primary endpoint.

### Statistical analysis

The study report will be prepared according to the STROBE (Strengthening the Reporting of Observational Studies in Epidemiology) reporting guidelines for observational studies^20^. CONSORT (Consolidated Standards of Reporting Trials) will be used to monitor data completeness from eligibility screening, approach, study acceptance and final follow-up. Analyses will be performed according to the statistical analysis plan. All data analysis will occur centrally and will be led by the study steering group.

Simple summary statistics will be calculated to describe demographic, procedure and outcome data, overall and at the individual site level. Data will be tested for distribution and differences between groups using unpaired *t*-tests, Mann–Whitney *U* tests and *χ* ^2^ tests, as appropriate. Kaplan–Meier survival analysis will be performed at the two study time points (3 and 5 years after the diagnosis of LRR). Cox proportional hazards multivariate regression analysis and log-rank tests for univariate analysis will be performed to include potential prognostic factors as variables of interest.

### Data validation and management

Data collection will occur in accordance with Good Clinical Practice (GCP), Caldicott principles and the General Data Protection Regulation (GDPR) 2018 and will work in line with NHS confidentiality guidelines and codes of conduct. Data for each patient will be pseudonymised using a unique alphanumeric study identification number. No patient identifiers other than the study number will be recorded in the REDCap database (the eCRF for the study). The local PI will keep a secure record of the RedCap ID with the corresponding NHS number (in England and Wales), Community Health Index number (in Scotland), or Health and Care number (in Northern Ireland). This is required for the research team at each participating centres to access the hospital electronic system at 3 and 5 years after LRR diagnosis to record oncological outcomes.

### Patient and public involvement

Cancer recurrence and long-term outcomes of treatment were key themes that emerged from the National Cancer Research Institute’s ‘living with and beyond cancer research priorities’ project https://www.ncri.org.uk/lwbc/, which surveyed over 3500 patients, carers and health and social care professionals. Furthermore, a James Lind Alliance priority setting partnership in breast surgery identified breast cancer recurrence as one of top 10 research priorities by both patients and healthcare professionals^21^. We have gained valuable input from a patient representative group (Independent Cancer Patients’ Voice, ICPV; PF, as a representative of the ICPV, is a member of the study steering group), especially in drafting the PIS and the consent form. Patient involvement will play a key part in design of the data analysis and dissemination plans.

## Discussion

The MARECA study is the first in the UK, which aims to determine the current management of breast cancer LRR across participating sites, explore investigations utilised, study cancer progression and treatment patterns, as well as potential variations in patient management. The MARECA study NPQ result^14^ has already demonstrated variation in certain aspects of patient management as stated by the participating breast MDTs. The planned sample size would create the largest prospective study cohort of patients diagnosed with LRR in the UK.

There are current uncertainties in the management of patients diagnosed with LRR. Patients may be classified as having a TR or a NP breast cancer. This classification is not routinely performed across UK breast units as its clinical implications remain uncertain. The collation of this study cohort enables the opportunity to use existing clinical classifications, determine what proportion of patients are diagnosed with a TR as opposed to a NP, and explore if these clinical classifications predict the patient’s subsequent prognosis. In addition, the uptake rate of the optional consent to donate archived tissue will facilitate the design of the tissue sub-study, which aims to refine the distinction between a TR and a NP, using molecular assays. The combined clinical and molecular information will enable further stratification of breast cancer LRR with an aim of delivering a more tailored treatment whilst optimising survival outcome.

The study will describe what proportion of patients diagnosed with LRR undergo radiological staging investigations, which imaging modalities are utilised, and the proportion of patients found to have associated DM at presentation. The study will explore and aim to identify a patient subgroup where routine staging investigations may not be required given the low pick-up rate of DM, with associated cost benefits to the healthcare system.

The study aims to provide data on which patient could be offered repeat BCS for LRR, how many patients are currently managed with repeat BCS, and how their outcomes compare against those undergoing mastectomy for LRR. The data from the MARECA study may inform the design of future trials exploring the role of repeat BCS for LRR. A repeat sentinel lymph node biopsy is offered when patients are diagnosed with LRR with a normal preoperative clinical assessment of the ipsilateral axillary lymph nodes. However, the methods of SLN relocalisation, the success rate of SLN reidentification and its long-term prognostic impact at the national level remains unexplored. The MARECA study is designed to gather such information, which will be valuable in setting future guidelines.

LRR may be due to the survival and proliferation of clones that are resistant to a range of adjuvant therapies used to treat primary breast cancer. Therefore, it is important to examine the choice of endocrine and/or chemotherapy in patients with LRR. CALOR trial^22^ demonstrated that patients with ER-negative LRR benefit from chemotherapy, but those with ER-positive LRRs do not. However, compared to treatment for primary breast cancer, there is a relative lack of clinical trial data on the efficacy of further adjuvant systemic therapy in patients diagnosed with LRR. The MARECA study will therefore gather data on the type of adjuvant treatments currently used to treat patients diagnosed with LRR in the UK.

There are potential limitations to the study’s design. The study is recruiting patients from UK breast units with varying number of breast cancer patients treated in each participating centres. Therefore, some units may be better represented than others. It is also unknown whether the COVID-19 pandemic and the required alterations in the management of primary breast cancer^23^, may impact on how patients are presenting with LRR, and whether there is a delay in diagnosis with a higher rate of associated DM. Lastly, it is also uncertain whether the patient uptake rate to the study may be higher in those found to have isolated LRR, as opposed to LRR with associated DM. There may be less inclination by the clinical team to approach the latter patient group, or these patients may be less willing to participate due to the noncurative nature of their disease. We also currently do not know the differential prognosis or the proportion of LRR patients diagnosed with different tumour subtypes. It maybe that based on the results of the 5-year survival analysis, an ethics amendment would be considered to follow-up particular subgroup (e.g. lobular breast cancer) of the study cohort for a longer period of time.

In conclusion, this prospective, multicentre cohort study will examine the current management and outcomes of patients diagnosed with breast cancer LRR in the UK. The resulting data will inform future research and support the development of best practice guidelines for this patient group.

## Study status

The MARECA study opened to patients in January 2022. The date for completion of study recruitment is 31/12/2023. The latest approved version of the study protocol is 4.0, dated 25.5.2022, approved on 20/06/2022 by the London - Brighton & Sussex Research Ethics Committee. As of 11/12/23, the study has recruited 684 patients and hence exceeded the minimum recruitment target of 500 patients.

## Ethics approval and consent to participate

This study was approved by London - Brighton & Sussex Research Ethics Committee on 07/09/2021 and by HRA on 08/09/2021 (21/PR/1128).

## Contact for public enquiries

Leedsth-tr.themarecastudy@nhs.net

## Consent

Written informed consent was obtained from patients.

## Sources of funding

The study was funded by the Association of Breast Surgery research development grant. The study also received funding from the Leeds Hospitals Charity. The funders had no role in the design of the study, data collection, analysis, interpretation of data, and in the writing of this manuscript.

## Author contribution

S.M.H.: wrote the first draft of the manuscript; S.M.H., J.L.M., V.WTC., P.A.B., E.C., R.I.C., R.D., B.E., P.F., B.H., K.H., C.C.K., S.A.M., R.L.O.C., N.P., S.P., T.R., L.S., L.W., and B.K.: all contributed to the study design, including design of the data collection instruments, and to the writing and editing of the protocol; S.M.H., E.C., R.I.C., R.D., P.F., B.H., K.H., C.C.K., S.M.cI., R.L.O.C., S.P., T.R., L.W., and B.K.: were responsible for the conception of the project, and contributed to the design of the project, and to the writing and editing of the protocol. All authors have read and approved the final version of the manuscript.

## Author statement

All persons who meet authorship criteria are listed as authors, and all authors certify that they have participated sufficiently in the work to take public responsibility for the content, including participation in the concept, design, analysis, writing, or revision of the manuscript.

## Conflicts of interest disclosures

SMcI reports honoraria from MSD, Roche, BD and Lilly, conference travel and support from Roche and Lilly, and institutional research funding from Novartis. RIC declares institutional research support from SECA and Astra Zeneca. EC reports honorarium from AstraZeneca, Eli Lilly, Novartis, Pfizer, Roche, Conference fees/travel/accommodation: Roche, Novartis, and Educational grant: Daiichi-Sankyo and Research support: SECA, AstraZeneca. All other authors declare that they have no competing interests.

## Research registration unique identifying number (UIN)

Trial registration: ISRCTN: Registered 25/11/2021, https://doi.org/10.1186/ISRCTN11908093

## Guarantor

Mr Baek Kim.

## Supplementary Material

## References

[1] professional/cancer-statistics/statistics-by-cancer-type/breast-cancerhwcoh.

[2] Marmot M Altman G Cameron DA . Independent UK panel on breast cancer screening replies to Michael Baum. BMJ 2013;346:f873. 23407732 10.1136/bmj.f873

[3] Shenouda MN Sadek BT Goldberg SI . Clinical outcome of isolated locoregional recurrence in patients with breast cancer according to their primary local treatment. Clin Breast Cancer 2014;14:198–204. 24485702 10.1016/j.clbc.2013.12.007

[4] Ebctcg McGale P Taylor C Correa C . Effect of radiotherapy after mastectomy and axillary surgery on 10-year recurrence and 20-year breast cancer mortality: meta-analysis of individual patient data for 8135 women in 22 randomised trials. Lancet 2014;383:2127–2135. 24656685 10.1016/S0140-6736(14)60488-8 PMC5015598

[5] Early Breast Cancer Trialists’ Collaborative G Darby S McGale P Correa C . Effect of radiotherapy after breast-conserving surgery on 10-year recurrence and 15-year breast cancer death: meta-analysis of individual patient data for 10,801 women in 17 randomised trials. Lancet 2011;378:1707–1716. 22019144 10.1016/S0140-6736(11)61629-2 PMC3254252

[6] Sirohi B Leary A Johnston SR . Ipsilateral breast tumor recurrence: is there any evidence for benefit of further systemic therapy? Breast J 2009;15:268–278. 19645782 10.1111/j.1524-4741.2009.00716.x

[7] Wong SM Golshan M . Management of in-breast tumor recurrence. Ann Surg Oncol 2018;25:2846–2851. 29947005 10.1245/s10434-018-6605-4

[8] Laird J Lok B Siu C . Impact of an in situ component on outcome after in-breast tumor recurrence in patients treated with breast-conserving therapy. Ann Surg Oncol 2018;25:154–163. 29094250 10.1245/s10434-017-6209-4 PMC5827945

[9] Yi M Buchholz TA Meric-Bernstam F . Classification of ipsilateral breast tumor recurrences after breast conservation therapy can predict patient prognosis and facilitate treatment planning. Ann Surg 2011;253:572–579. 21209588 10.1097/SLA.0b013e318208fc2a PMC4331097

[10] Lips EH Kumar T Megalios A . Genomic analysis defines clonal relationships of ductal carcinoma in situ and recurrent invasive breast cancer. Nature Genet 2022;54:850–860. 35681052 10.1038/s41588-022-01082-3 PMC9197769

[11] Bollet MA Servant N Neuvial P . High-resolution mapping of DNA breakpoints to define true recurrences among ipsilateral breast cancers. J Nationl Cancer Instit 2008;100:48–58. 10.1093/jnci/djm266 18159071

[12] Coles CE Griffin CL Kirby AM . Partial-breast radiotherapy after breast conservation surgery for patients with early breast cancer (UK IMPORT LOW trial): 5-year results from a multicentre, randomised, controlled, phase 3, non-inferiority trial. Lancet 2017;390:1048–1060. 28779963 10.1016/S0140-6736(17)31145-5 PMC5594247

[13] Murray Brunt A Haviland JS Wheatley DA . Hypofractionated breast radiotherapy for 1 week versus 3 weeks (FAST-Forward): 5-year efficacy and late normal tissue effects results from a multicentre, non-inferiority, randomised, phase 3 trial. Lancet 2020;395:1613–1626. 32580883 10.1016/S0140-6736(20)30932-6 PMC7262592

[14] Morgan JL Cheng V Barry PA . The MARECA (national study of management of breast cancer locoregional recurrence and oncological outcomes) study: National practice questionnaire of United Kingdom multi-disciplinary decision making. Eur J Surg Oncol 2022;48:1510–1519. 35410760 10.1016/j.ejso.2022.03.017

[15] Potter S Conroy EJ Cutress RI . Short-term safety outcomes of mastectomy and immediate implant-based breast reconstruction with and without mesh (iBRA): a multicentre, prospective cohort study. Lancet Oncol 2019;20:254–266. 30639093 10.1016/S1470-2045(18)30781-2 PMC6358590

[16] O’Connell RL Baker E Trickey A . Current practice and short-term outcomes of therapeutic mammaplasty in the international TeaM multicentre prospective cohort study. Br J Surg 2018;105:1778–1792. 30132807 10.1002/bjs.10959

[17] Whitehead I Irwin GW Bannon F . The NeST (Neoadjuvant systemic therapy in breast cancer) study: National Practice Questionnaire of United Kingdom multi-disciplinary decision making. BMC Cancer 2021;21:90. 33482770 10.1186/s12885-020-07757-6 PMC7825231

[18] Harris PA Taylor R Thielke R . Research electronic data capture (REDCap)–a metadata-driven methodology and workflow process for providing translational research informatics support. J Biomed Inform 2009;42:377–381. 18929686 10.1016/j.jbi.2008.08.010 PMC2700030

[19] Harris PA Taylor R Minor BL . The REDCap consortium: building an international community of software platform partners. J Biomed Inform 2019;95:103208. 31078660 10.1016/j.jbi.2019.103208 PMC7254481

[20] Vaidya JS Massarut S Vaidya HJ . Rethinking neoadjuvant chemotherapy for breast cancer. BMJ 2018;360:j5913. 29326104 10.1136/bmj.j5913

[21] Potter S Fairhurst K Cowan K . Identifying research priorities in breast cancer surgery: a UK priority setting partnership with the James Lind Alliance. Breast Cancer Res Treat 2023;197:39–49. 36319906 10.1007/s10549-022-06756-4 PMC9628302

[22] Wapnir IL Price KN Anderson SJ . Efficacy of chemotherapy for ER-Negative and ER-Positive isolated locoregional recurrence of breast cancer: final analysis of the CALOR Trial. J Clin Oncol 2018;36:1073–1079. 29443653 10.1200/JCO.2017.76.5719 PMC5891132

[23] Courtney A O’Connell R Rattay T . The B-MaP-C study: breast cancer management pathways during the COVID-19 pandemic. study protocol. Int J Surg Protoc 2020;24:1–5. 32838092 10.1016/j.isjp.2020.07.003 PMC7388760

