# Supplementary material for: The MARECA (national study of management of breast cancer locoregional recurrence and oncological outcomes) study: protocol for a prospective, multicentre cohort study
Source: Int J Surg Protoc. 2024 Jan 26;28(1):20–6. doi: 10.1097/SP9.0000000000000018 (PMC10905491; doi:10.1097/SP9.0000000000000018)
Supplement: Supplementary file 1 [file sp9-28-20-s001.pdf]

CASE REPORT FORM

The MARECA study: National study of management of breast cancer locoregional recurrence and oncological outcome

Eligibility check:

**Inclusion criteria (tick box)**	
Age ≥ 18yrs ☐ Recently (within the last 6 months) diagnosed with new ipsilateral breast cancer locoregional recurrence +/- distant metastasis	☐
Treated for previous unilateral or bilateral breast cancer with curative intent	☐
No previous evidence of distant metastatic disease	☐
Able to provide informed consent	☐
Minimum of 3 months interval between the resection surgery for the original cancer and the diagnosis of locoregional recurrence (no maximum interval time period)	☐

**Exclusion criteria (tick box) If any of boxes are ticked, the patient is ineligible**	
Patients where the new breast cancer is in the contralateral breast	☐
Patients diagnosed with distant metastasis with no evidence of locoregional recurrence	☐
Patients diagnosed with angiosarcoma	☐
Patients with a history of non-breast cancer which was non-curative in intent	☐
Previous ipsilateral surgery for atypia, benign conditions, or phyllodes tumour and other breast sarcomas AND no previous ipsilateral primary breast cancer resection	☐

Entry Details

**Centre Name**	
**Study. No**	
**Name of person taking consent:**	
**Date of consent**	
**Optional consent for tissue/slide access**	**Yes / No**

The following data regarding the patient's original and recurrent cancer can be collected once eligible patients have provided written consent. Please upload the data onto the REDCap database.

Baseline Demographics (circle answers)

Patient age: Yrs

Body mass index:

Current Menopausal Status

1 = pre-menopausal 2 = post-menopausal 3 = peri-menopausal

Smoking status: Non-smoker Ex-smoker Current smoker

ECOG performance status (0-4): 0 1 2 3 4

Family History: unknown population risk moderate risk high risk BRCA positive other known breast cancer gene mutation (e.g. TP53)

Surgery data - original cancer (circle answers)

Date of original breast cancer surgery:

Patient age at original diagnosis:

Presentation: screen-detected symptomatic

Type of surgery: Breast conserving surgery Mastectomy + immediate reconstruction

Mastectomy + delayed reconstruction

Mastectomy + no reconstruction No breast surgery

If breast conserving surgery for invasive lobular cancer, was MRI performed: Yes / No / N/A

The original tumour location: Upper inner quadrant Upper outer quadrant

Lower inner quadrant Lower outer quadrant

Subareolar or central

Type of axilla surgery: SLNB ANC ANS targeted axillary dissection

No axillary surgery

If SLNB, method of localisation: Dual technique with blue dye and radioisotope

Radioisotope alone

Blue dye alone

Other methods

Was the original cancer inflammatory breast cancer: Yes / No

Pathological Data - original cancer

Tumour subtype: Invasive ductal Invasive lobular Mixed ductal/lobular
Other subtypes Pure DCIS

Tumour grade: 1 2 3 (if invasive)
Low Intermediate High (If DCIS)

Tumour size: …… mm (invasive tumour size, unless DCIS)

Multifocal or multicentric cancer: Yes No

Lymphovascular invasion: Yes No

Closest radial margin after final surgery: …… mm

Were anterior or posterior margins close (1mm or less)? Yes No

If Yes, please state which (anterior / posterior / both) and distance in mm: ….. mm

State the number of positive axillary nodes: …..

Please circle whether macrometastasis or micrometastasis

Please state the number of nodes retrieved: …..

ER receptor Allred score: 0 1 2 3 4 5 6 7 8 (please state if any other scoring system was used and its score; e.g. H-score; ……………)

PR receptor Allred score: 0 1 2 3 4 5 6 7 8 (please state if any other scoring system was used and its score; e.g. H-score; ……………)

Her2 receptor: Positive Negative

Please state immunohistochemistry score 0 1+ 2+ 3+

If 2+. was FISH test done? What was the final HER2 receptor status if FISH test done; positive/negative/not applicable

Ki67 (if performed, please state percentage): …… %

Adjuvant Treatments - original cancer (circle answers)

Radiotherapy post-BCS: Yes No N/A
Radiotherapy post-mastectomy: Yes No N/A
Radiotherapy to the axilla: Yes No
Radiotherapy to SCF: Yes No
Radiotherapy to IMN: Yes No

If radiotherapy after BCS, state regimen:

WBRT; 40 Gy 15 fractions WBRT; 27 Gy 5 fractions (hypofractionated)
Partial breast radiotherapy WBRT with boost

Endocrine Therapy: Yes No (If Yes, state total duration received: ….. Yrs)
Please state 1st line endocrine therapy: Tamoxifen Letrozole Anastrozole Exemestane Ovarian supression Others
Please state duration: ….. Yrs

Please state 2nd line endocrine therapy (if applicable): Tamoxifen Letrozole Anastrozole Exemestane Ovarian supression Others
Please state duration: ….. Yrs (if applicable)

Chemotherapy: Yes No (If Yes, circle Adjuvant / Neoadjuvant)

Usage of Oncotype Dx or other predictive test: Yes No
If Yes, please state test used and score: ………………………

Please select which chemotherapy agents the patient received:
Epirubicin / Doxorubicin / Cyclophosphamide / Docetaxel / Paclitaxel / Carboplatin / Capecitabine / Others; please state

Please state if chemotherapy was stopped early: Yes No

Anti-HER2 therapy: Yes No
If Yes, please state regimen: **Trastuzumab (Herceptin)**
Trastuzumab (Herceptin) + Pertuzumab (Perjeta)

Bisphosphonates: Yes No

Locoregional recurrence treatment details (circle answers)

Diagnosis

Date of locoregional recurrence diagnosis: **Age at diagnosis: ….. Yrs**

Presentation: screen-detected symptomatic

Tumour subtype: Invasive ductal Invasive lobular Mixed ductal/lobular

Other subtypes Pure DCIS

If invasive cancer diagnosis, was there associated DCIS: Yes No

Tumour grade: 1 2 3 (if invasive)

Low Intermediate High (If DCIS)

ER receptor Allred score: 0 1 2 3 4 5 6 7 8 (please state if any other scoring system was used and its score; e.g. H-score; ……………)

PR receptor Allred score: 0 1 2 3 4 5 6 7 8 (please state if any other scoring system was used and its score; e.g. H-score; ……………)

Her2 receptor: Positive Negative

Please state immunohistochemistry score 0 1+ 2+ 3+

If 2+. was FISH test done? What was the final HER2 receptor status if FISH test done; positive/negative/not applicable

Ki67 (if performed, please state percentage): …… %

If patient had BCS for the original cancer, please state the tumour location of the recurrent cancer:

Upper inner quadrant Upper outer quadrant

Lower inner quadrant Lower outer quadrant

Subareolar or central N/A

Radiology:

Site(s) of locoregional recurrence (please circle all that applies):

breast / skin / chest wall / axilla / internal mammary nodes / supraclavicular nodes / infraclavicular nodes

If breast LRR, state the maximal tumour size on imaging: ….. mm

Axilla ultrasound scan: Yes No

If yes, was it reported as normal: Yes No

If reported as abnormal, what was the FNA/core biopsy result:

C1 C2 C3 C4 C5

B1 B2 B3 B4 B5

Staging investigation Yes No

If Yes, please circle modality used: CT CT + Bone scan PET CT Others

Did staging reveal distant metastasis Yes No

If Yes, please circle site(s): Bone Brain Liver Lung Other sites

Was cancer resection surgery performed in the presence of distant metastasis: Yes No

Was Neoadjuvant systematic therapy used: No NACT NAET CDK inhibitors Others

Surgery for locoregional recurrence:

Surgery for locoregional recurrence Yes No (**If Yes, please state date:**)

If no surgery for locoregional recurrence, please state reason;

Unresectable Patient unfit for surgery Patient choice Concurrent distant metastasis

Commenced systemic therapy first Other reasons (please specify)

Type of breast surgery: None Simple mastectomy Repeat BCS

WLE of skin flap or chest wall recurrence (direct closure / skin graft / flap closure)

Mastectomy with deconstruction (taking down previous recon)

Mastectomy with IBR: Implant Fully autologous Autologous + implant

Planned axillary surgery: SLNB (no previous axillary surgery) Repeat SLNB

ANS ANC TAD None

Axillary exploration after previous ANC Others

If repeat SLNB, method of localisation: Dual technique with blue dye and radioisotope

Radioisotope alone

Blue dye alone

Other methods

If repeat SLNB, was the sentinel lymph node identified: Yes No

If repeat SLNB failed to identify the sentinel lymph node, how did you proceed:

No further axillary dissection ANS ANC

Further pathology on locoregional recurrence:

Multifocal or multicentric cancer: Yes No

Lymphovascular invasion: Yes No

Tumour size: …… mm (invasive size, unless DCIS)

Closest radial margin: …… mm

Were anterior or posterior margins close (1mm or less)? Yes No

If Yes, please state which (anterior / posterior / both) and distance in mm: ….. mm

State the number of positive axillary nodes: …..

Please circle whether macrometastasis or micrometastasis

Please state the number of nodes retrieved: …..

Further locoregional and systemic treatment (including further surgery)

Further surgery to the breast Yes No
If Yes, please state the type of further surgery Margin re-excision Mastectomy

Further axillary surgery Yes No
If Yes, please state the type of further surgery ANC SLNB ANS

Receipt of breast radiotherapy Yes No N/A

If radiotherapy after BCS, please state regimen:
WBRT; 40 Gy 15 fractions WBRT; 27 Gy 5 fractions (hypofractionated)
Partial breast radiotherapy WBRT with boost

Radiotherapy post-mastectomy: Yes No N/A
Radiotherapy to the axilla: Yes No
Radiotherapy to SCF: Yes No
Radiotherapy to IMN: Yes No

Endocrine Therapy: Yes No (If Yes, state duration recommended at MDT: ….. Yrs)
If Yes, please state the prescribed endocrine therapy: Tamoxifen Letrozole Anastrozole

Exemestane Ovarian supression Others

Chemotherapy: Yes No

Please select which chemotherapy agents the patient received:
Epirubicin / Doxorubicin / Cyclophosphamide / Docetaxel / Paclitaxel / Carboplatin / Capecitabine / Others; please state

Anti-HER2 therapy: Yes No

If Yes, please state regimen: **Trastuzumab (Herceptin)**
Trastuzumab (Herceptin) + Pertuzumab (Perjeta)

Other targeted treatment Yes No

If Yes, please specify

3 and 5 year data collection

Patient alive: Yes No

If Death, was it related to breast cancer: Yes No

If no, please state the cause of death:

Please state the date of death:

Any further locoregional recurrence +/- distant metastasis Yes No

Please state the date of further locoregional recurrence:

Please state the date of distant metastasis:

Full details will require uploading to REDCap
